# Supplementary figures and images for: Electrical writing, deleting, reading, and moving of magnetic skyrmioniums in a racetrack device
Source: Sci Rep. 2019 Aug 20;9:12119. doi: 10.1038/s41598-019-48617-z (PMC6702348; doi:10.1038/s41598-019-48617-z)

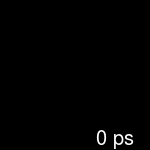

Supplement: Supplementary file 1 — Supplementary Video 1 [file 41598_2019_48617_MOESM1_ESM.gif]

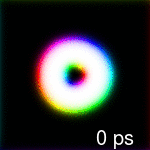

Supplement: Supplementary file 2 — Supplementary Video 2 [file 41598_2019_48617_MOESM2_ESM.gif]

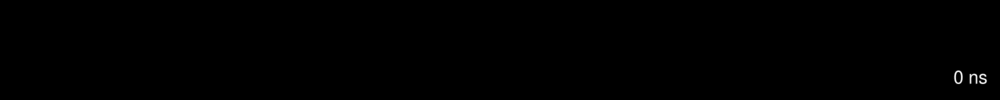

Supplement: Supplementary file 3 — Supplementary Video 3 [file 41598_2019_48617_MOESM3_ESM.gif]

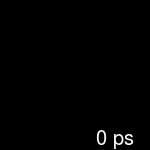

Supplement: Supplementary file 4 — Supplementary Video 4 [file 41598_2019_48617_MOESM4_ESM.gif]

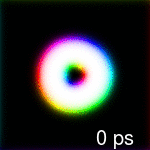

Supplement: Supplementary file 5 — Supplementary Video 5 [file 41598_2019_48617_MOESM5_ESM.gif]

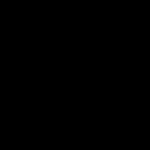

Supplement: Supplementary file 6 — Supplementary Video 6 [file 41598_2019_48617_MOESM6_ESM.gif]

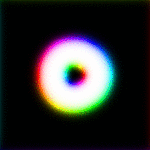

Supplement: Supplementary file 7 — Supplementary Video 7 [file 41598_2019_48617_MOESM7_ESM.gif]
