# Supplementary material for: Electrical writing, deleting, reading, and moving of magnetic skyrmioniums in a racetrack device
Source: Sci Rep. 2019 Aug 20;9:12119. doi: 10.1038/s41598-019-48617-z (PMC6702348; doi:10.1038/s41598-019-48617-z)
Supplement: Supplementary file 8 — Supplementary information [file 41598_2019_48617_MOESM8_ESM.pdf]

Electrical writing, deleting, reading, and moving of magnetic skyrmioniums in a racetrack device — Supplementary information —

Börge Göbel,[1] Alexander F. Schäffer,[2] Jamal Berakdar,[2] Ingrid Mertig,[1,2] and Stuart S. P. Parkin[1]

[1]*Max-Planck-Institut für Mikrostrukturphysik, D-06120 Halle (Saale), Germany*

[2]*Institut für Physik, Martin-Luther-Universität Halle-Wittenberg, D-06099 Halle (Saale), Germany*

In this Supplementary information we show five additional figures which were mentioned in the main paper. In Supplementary Fig. 1 we show the skyrmionium-skyrmionium interaction potential. In Supplementary Fig. 2 the current dependence of the skyrmionium velocity is analyzed. Supplementary Fig. 3 visualizes an optoelectrically generated skyrmionium at room temperature. In Supplementary Fig. 4 we analyze the skyrmionium generation process for different writing parameters. Finally, in Supplementary Fig. 5 the skyrmionium stability and current-driven motion are shown for different DMI constants. More information is given in the caption of the respective figure.

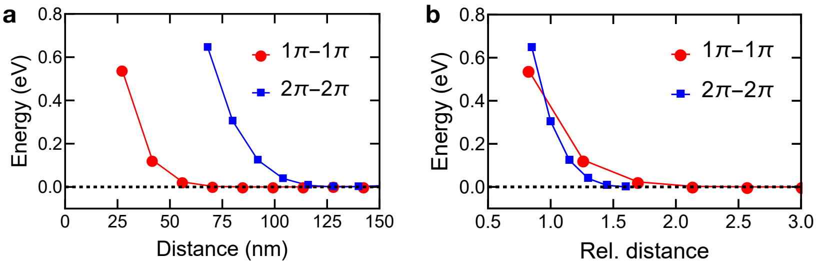

Supplementary Fig. 1: **Repulsive potential energies between magnetic quasiparticles.** The skyrmion-skyrmion ($1\pi - 1\pi$) and skyrmionium-skyrmionium ($2\pi - 2\pi$) interaction energies have been extracted from micromagnetic simulations of an unconfined system (periodic boundary conditions in the plane). The distance dependence is obtained from pinning the centers of the two interacting quasiparticles at different distances and subsequent relaxation of the magnetization configuration. **a** For both objects, the potential decays rapidly for distances larger than the intrinsic size of the objects (skyrmion: $\approx 33\,\mathrm{nm}$; skyrmionium: $\approx 80\,\mathrm{nm}$). **b** As panel a but the distance is normalized by the size of the respective particles.

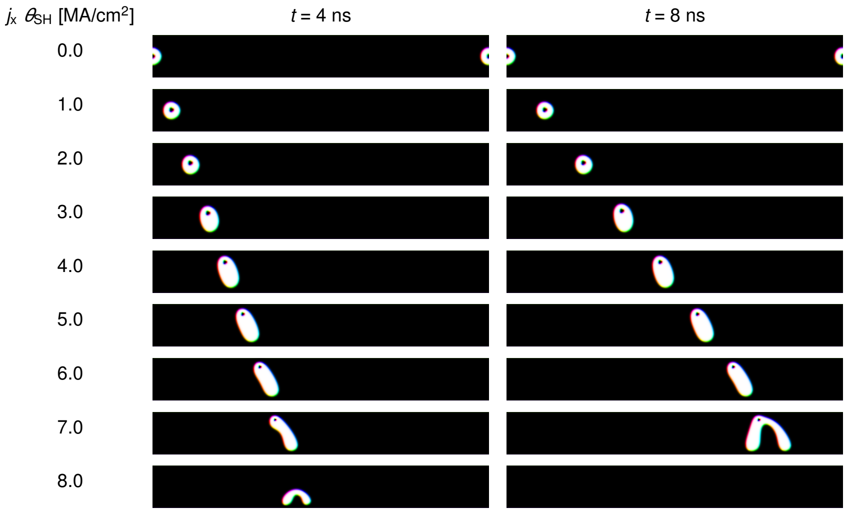

Supplementary Fig. 2: **Current dependence of skyrmionium velocity.** The left column indicates the simulated configuration after $4\,\mathrm{ns}$ and the right column after $8\,\mathrm{ns}$ of propagation time. The applied current density increases linearly from the top to the bottom row. The skyrmionium velocity increases nearly linearly until an effective current density $j_x\theta_{\mathrm{SH}}$ of around $6\,\mathrm{MA/cm^2}$ is reached. The skyrmionium velocity is approximately $140\,\mathrm{m/s}$ in this case. For higher values the skyrmionium strongly deforms, transitions to a skyrmion and annihilates. A utilization in the context of a racetrack storage is not possible anymore. The simulated racetrack has the dimensions $1600\,\mathrm{nm} \times 200\,\mathrm{nm} \times 1\,\mathrm{nm}$ with periodic boundary conditions along the racetrack direction.

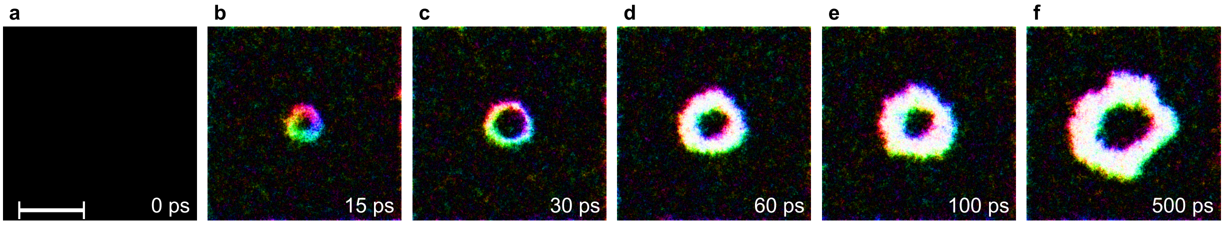

Supplementary Fig. 3: **Skyrmionium generation at room temperature.** Similar to Fig. 2 of the paper but for $T = 300\,\mathrm{K}$. The system size is increased to an area of $300\,\mathrm{nm} \times 300\,\mathrm{nm} \times 1\,\mathrm{nm}$ to show the stability of the skyrmionium without an additional stabilization by the boundary. All other parameters are equal to the ones used in the main text. The scale bar corresponds to $100\,\mathrm{nm}$.

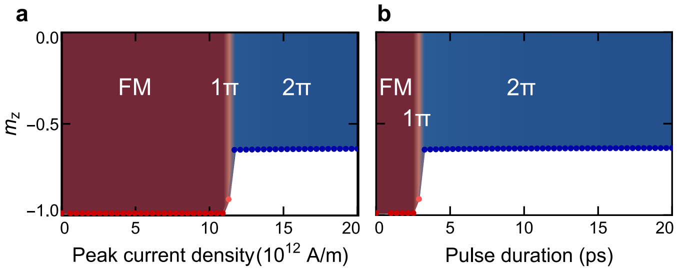

Supplementary Fig. 4: **Analysis of the writing parameters.** Resulting spin configuration in dependence of **a** the peak current density and **b** the pulse duration. The final state is characterized by the averaged z component of the magnetization, indicating a collinear ferromagnetic state (FM), a skyrmion (1π) or a skyrmionium (2π). Due to the different sizes of skyrmion and skyrmionium, the net magnetization allows to distinguish them. For both writing parameters a threshold needs to be overcome to reliably create skyrmioniums. For **a** the FWHM pulse duration is fixed to $9\,\mathrm{ps}$, **b** assumes a fixed peak current density of $2 \times 10^{13}\,\mathrm{A\,m^{-1}}$. Both values correspond to the parameters used in the paper.

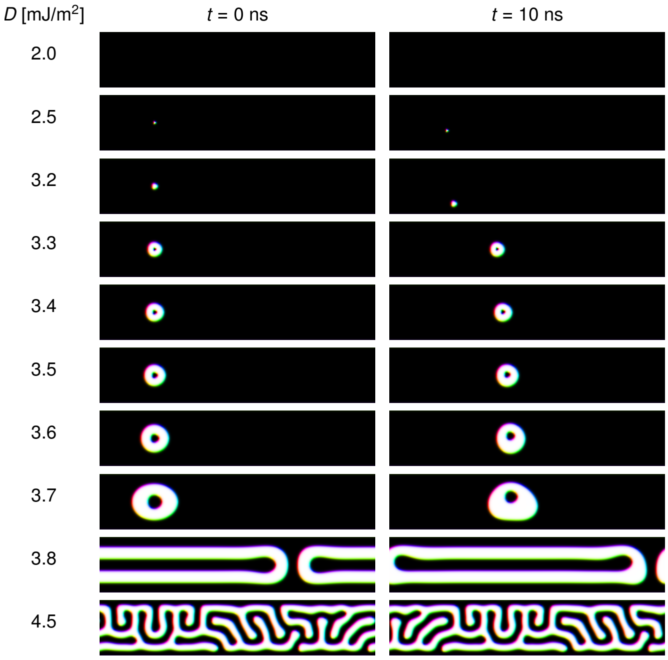

Supplementary Fig. 5: **DMI dependence of skyrmionium stability and current-driven motion.** The left column shows the relaxed texture and the right column shows the texture after a propagation for $10\,\mathrm{ns}$ under the influence of an effective current density of $j_x\theta_{\mathrm{SH}} = 1\,\mathrm{MA/cm^2}$. The DMI constant D increases from the top to the bottom row as indicated. The skyrmionium is stable for D between $3.3\,\mathrm{mJ/m^2}$ and $3.7\,\mathrm{mJ/m^2}$. The simulated racetrack has the dimensions $1000\,\mathrm{nm} \times 200\,\mathrm{nm} \times 1\,\mathrm{nm}$ with periodic boundary conditions along the racetrack direction.
